# Pulmonary arterial hypertension in adult onset Still’s disease: a case report of a severe complication

**DOI:** 10.1186/s12890-016-0237-x

**Published:** 2016-05-10

**Authors:** L. Guilleminault, S. Laurent, A. Foucher, P. Poubeau, F. Paganin

**Affiliations:** Service de Pneumologie, CHU Reunion/GHSR, Saint-Pierre, F-97410 France; Inserm, UMR 1188 Diabète athérothrombose Thérapies Réunion Océan Indien (DéTROI), plateforme CYROI, Sainte-Clotilde, F-97490 France; Université de La Réunion, UMR 1188, Sainte-Clotilde, F-97490 France; Service de maladies infectieuses, CHU Reunion/GHSR, Saint-Pierre, F-97410 France; Service de Pneumologie, Groupe Hospitalier Sud Réunion, BP 350, 97448 Saint-Pierre cedex, France

**Keywords:** Case report, Pulmonary arterial hypertension, Adult onset Still’s disease, Auto-inflammatory disease

## Abstract

**Background:**

Adult onset of Still’s disease (AOSD) is a rare systemic inflammatory disease. Cardiorespiratory complications are mainly represented by pleural and pericardial disorders and are less frequent than cutaneous and articular complaints. Pulmonary arterial hypertension (PAH) occurring in AOSD is rarely described in literature.

**Case presentation:**

We present the case of a young patient who developed severe PAH 2 years after diagnosis of AOSD. This is a rare and severe complication which is probably underestimated.

**Conclusions:**

PAH in AOSD can be lethal, and unfortunately its occurrence is unpredictable. Echocardiographic screening of AOSD patients should be evaluated in further trials. Currently, the most suitable treatment is still unknown.

**Electronic supplementary material:**

The online version of this article (doi:10.1186/s12890-016-0237-x) contains supplementary material, which is available to authorized users.

## Background

Adult onset Still’s disease (AODS) is a rare auto-inflammatory disease presenting with a wide range of non-specific symptoms [1]. In most cases, patients have spiking fever, arthralgia, rash and high leukocytosis, which are major items of criteria described in the literature [2, 3]. Cardio-respiratory complications are mainly represented by pleural and pericardial disorders [4, 5]. We report herein the case of a young woman with AOSD who developed pulmonary hypertension. This complication is rarely observed but pathophysiology of this phenomenon is unkown.

## Case presentation

### Initial presentation and diagnosis

**Fig. 1:**
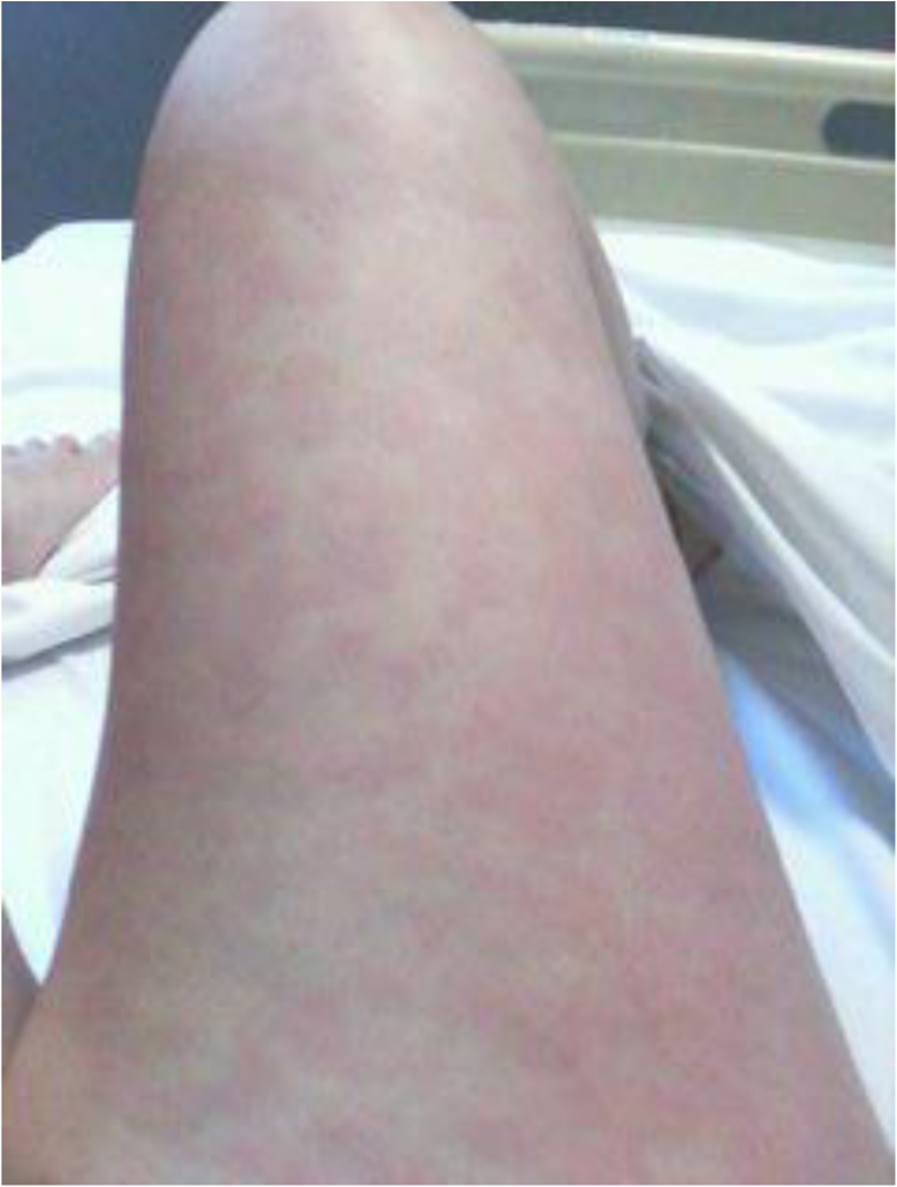
Evanescent urticaria-like rash on right leg in our patient with adult-onset Still’s disease

**Fig. 2:**
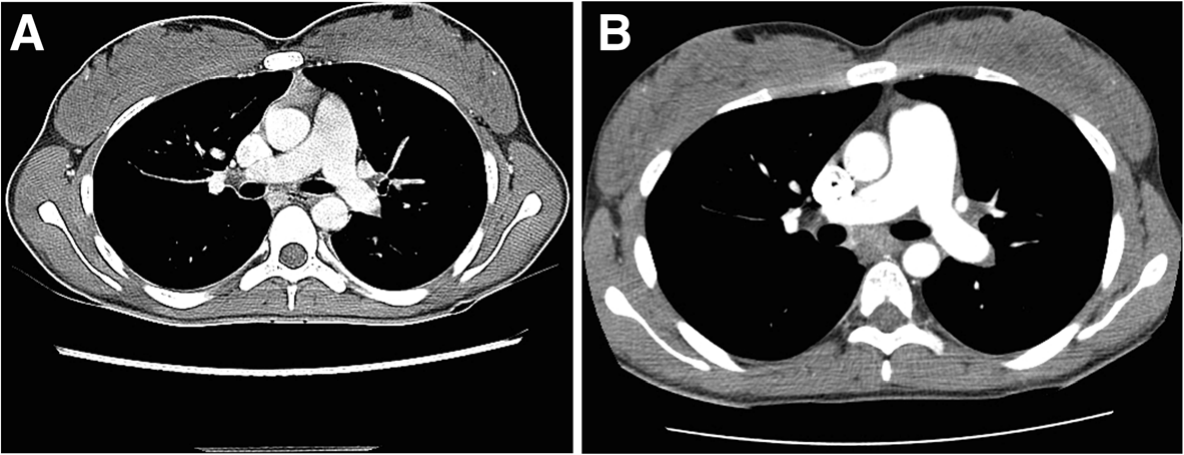
**a** CT scan performed at AOSD diagnosis with a pulmonary trunk (PT) at 21.7 mm, aorta trunk at 22.8 mm. PT/Aorta trunk index was 0.95. **b** CT scan performed 2 years after diagnosis with a pulmonary trunk (PT) at 31 mm, aorta trunk at 22.1 mm. PT/Aorta trunk index was 1.4

A 19 year-old female presented with persistent fever, evanescent rash (Fig. 1), sore throat and polyarthralgia without synovitis (bilateral knees, shoulders, elbows and metatarsophalangeal joints). Physical examination showed axillary and inguinal lymphadenopathy (diameter < 1 cm). Investigations revealed a high erythrocyte sedimentation rate (87 mm/first hour) and high C-reactive protein (CRP) (116 mg/L). Serum creatinine, liver enzymes, CPK, granulocytes and platelet levels were all normal. Rheumatoid factor, anti CCP, antinuclear antibodies and antineutrophil cytoplasm antibodies were negative. Blood cultures were negative. Serological tests for virus (EBV, CMV, HSV and HIV) were negative. Ferritin level was high (8140 μg/L) and glycosylated ferritin was low (rate: 9 %). Computed tomography (CT) scan of chest, abdomen and pelvis was normal (Fig. 2a). The presentation suggested a systemic rheumatic disease and the investigations excluded alternative auto-immune diagnoses. AOSD was finally proposed, as a diagnosis of exclusion, based on the association of arthritis, rash, fever, elevated acute phase reactants and low glycosylated ferritin.

### Treatment

She received oral steroids (1 mg/kg), which led to clinical and laboratory improvement. Symptoms reappeared during the tapering-off phase (25 mg.d^−1^) and the dose was increased again to 1 mg/kg with the addition of a 10 mg methotrexate dose once a week. Despite methotrexate therapy, the patient experienced a second relapse when the steroid dose was decreased (25 mg.d^−1^). Methotrexate dose was increased to 15 mg once per week, with a steroid dose at 25 mg.d^−1^. Decision was made to introduce anakinra (100 mg.d^−1^) as a steroid sparing agent. Steroid dosage was decreased and methotrexate was discontinued but subsequently reintroduced due to a reappearance of skin rash.

### Respiratory complication

Two years after AOSD diagnosis, the patient presented with NYHA class III dyspnea associated with polyarthritis, fatigue and ferritin increase. At that time, therapy was methotrexate (15 mg once per week), corticosteroids (25 mg.d^−1^) and anakinra. There was no sign of right-sided heart failure and the electrocardiogram was normal. The patient walked 462 m with oxygen desaturation (SpO2 : 87 %) on a 6-min walk test (6MWT). Vital capacity was 1.6 L (49 %), and total pulmonary capacity 2.44 L (59 %). Carbon monoxide diffusion capacity was low (22 %). We did not have any reference values as the patient had no previous pulmonary function tests available. Arterial blood gas analysis revealed hypoxemia (pO2 : 71 mm.Hg^−1^) and hypocapnia (pCO2 : 34 mm.Hg^−1^). BNP, troponin, serum uric acid, creatinin and CRP were normal. Chest HRCT showed a dilated pulmonary trunk (PT) (31 mm); the PT/Aorta trunk index was 1.4 and the right ventricle/left ventricle (RV/LV) index was 1.26 (Fig. 2b). There was no pleural or parenchymal involvement of the lungs; no signs of pulmonary vasculitis, and no pulmonary embolism as confirmed by a ventilation/perfusion scan. Doppler echocardiography showed a systolic pulmonary artery pressure of 65 mm.Hg^−1^. Pulmonary hypertension was confirmed by right heart catheterization with a mean pulmonary artery pressure (mPAP) of 33 mm.Hg^−1^ and normal PCWP (4 mm.Hg^−1^). The cardiac output was 4.0 L.mn^−1^ and pulmonary resistance was 7.25 UWood. A Nitric oxide test was negative.

### Outcome

The patient was included in a clinical trial and received ambrisentan and tadalafil associated with ongoing anakinra and corticosteroids. Methotrexate was removed to avoid adverse effects on the lungs or liver. Ambrisentan was rapidly discontinued due to cytolytic hepatitis. One month later, she was admitted to ICU for right-sided heart failure; a thrombotic thrombocytopenic purpura was observed (low platelets at 5G/L, anaemia at 6.7 g/dL, LDH: 905 UI/L ADAMTS13: 31 % with N: 50–150 %). Dobutamine and intraveinous (IV) prostacycline were administered, with a progressive improvement. The patient showed a clinical improvement with a distance of 696 m on 6MWT under IV protacycline, tadalafil, anakinra and corticosteroids. One month later, she was admitted again to intensive care for septic shock with a staphylococcus epidermidis infection. The tunneled catheter was removed and bacteriological analyses found staphylococcus epidermidis. Evolution was poor and she passed away despite antibiotic treatment (piperacillin/amikacin) and vasoactive drug support.

## Discussion

AOSD is a rare systemic autoinflammatory disease with a prevalence ranging from 1 to 34 cases per million people [6–8]. Young people are preferentially affected with a mean age at diagnosis around 36 years old [9, 10]. Clinical presentation is not specific and a wide range of symptoms may be observed [4]. A high white blood cell count is common but was not observed in our patient. No laboratory test is specific but a low glycosylated ferritin (<20 %) may be suggestive.

In a ten-year retrospective study, none of the 28 patients with an AOSD diagnosis died [11]. The median follow-up was 3.72 years. However, other studies report a mortality rate around 10 % [12, 13]. Life expectancy and quality of life in AOSD patients may deteriorate with some complications. Reactive hemophagocytic lymphohistiocytosis seems to be the most frequent complication (about 12–15 %) and is particularly associated with a high mortality rate [14]. Other complications such as pericarditis, pleuritis, disseminated intravascular coagulation, thrombotic microangiopathy, fulminant hepatitis and AA amyloidosis are rarely observed [15–19].

PAH is a very rare complication of AOSD [1]. Its prevalence is probably under-estimated as only symptomatic case reports have been documented. Only 9 cases of PAH in AOSD patients have been published in the literature (Additional file 1: Table S1) [20–27]. Age of onset varies from 18 to 38 years old. A female predominance was reported (8 females to 1 male). Onset of PAH ranges from 0 to 108 months after AOSD diagnosis with a median of 24 months, which is concordant with our case. PAH may be a life-threatening complication with a 40 % fatality in previously reported cases. However, it is possible that authors preferentially published fatal cases, therefore introducing a bias in the prognosis.

Pathophysiology of PAH with AOSD is unknown. AOSD is an autoinflammatory disease in which pathogenesis is dominated by neutrophil and macrophage (innate immunity cells) activation. Several non-specific cytokines and chemokines such as IL-1β, IL-6, IL-18 and TNFα are particularly involved in AOSD pathogenesis [28–30]. In idiopathic PAH (IPAH) or connective tissue disease associated with PAH (CTD-PAH), it is widely accepted that alteration of immune mechanisms plays a significant role by recruiting inflammatory cells, remodeling the pulmonary vasculature, and promoting autoimmune responses [31]. IL-1β, IL-6, IL-18 and other cytokines are increased in IPAH patients, reflecting a dysregulation of innate immunity, also observed in AOSD. Pulmonary microangiopathy, which is similar to renal involvement and thrombotic thrombocytopenic purpura (which was observed in our patient), could participate in pathogenesis of PAH with AOSD [25]. Further studies are needed to properly characterise the pathogenesis in order to classify PAH with AOSD in the Pulmonary Hypertension World Health Organisation (WHO) clinical classification system. Similarities with IPAH and CTD could lead to classification of AOSD PAH as group 1.

In systemic sclerosis, the high incidence and prevalence of PAH associated with a devastating evolution in untreated cases, led to the recommendation of systematic and regular echocardiography screening in this high risk population. In our opinion, echocardiography screening in AOSD could have a major impact: (1) to determine PAH prevalence in AOSD patients. (2) to evaluate real prognosis and specific mortality. (3) to include these patients in PAH treatment clinical trials.

Considering the major role of IL-1 in AOSD, Anakinra was used in several case reports of AOSD patients with PAH because of its favourable systemic and rheumatic effect. However, in previous case reports, 3 out of 4 patients treated with anakinra worsened. Vasodilator therapies could be an option but have to be evaluated. To our knowledge, no data are available about vasodilator effect in PAH with AOSD. Manipulation of a tunnelled catheter in a patient treated with immunosuppressant drugs increases the risk of infection and prostacycline use should be discussed. Only one case of PAH improvement after tocilizumab, an anti-IL-6 antibody, has been reported in the literature. IL-6 plays a key role in the pathophysiology of AOSD but also in PAH. Tocilizumab may be an option in this indication.

## Conclusion

In conclusion, PAH in AOSD is a rare but potentially life-threatening complication. Pathogenesis is unknown but similarities between AOSD and IPAH or CTD-PAH on innate immunity dysregulation may explain a part of the pathophysiology. Screening AOSD patients with echocardiography should be assessed to determine the impact of PAH. Anakinra is used as first line therapy after induction with corticosteroids. Tocilizumab may be used as a second line therapy. To the best of our knowledge, vasodilators have never been assessed in this indication. Clinical trials about biologics or vasodilator treatments are mandatory.

### Ethics of approval

Not applicable.

### Consent

Written informed consent was obtained from the permitted assigns for publication of this case report and any accompanying images. A copy of the written consent is available for review by the Editor of this journal.

### Availability of data and materials

Data are not available for obvious reasons of patient confidentiality.

## Additional file

Additional file 1: Table S1. Case reports of pulmonary arterial hypertension in adult onset Still’s disease in the literature. (DOC 35 kb)

## Abbreviations

6MWT: 6-min walk test
AOSD: Adult onset Still’s disease
CRP: c-reactive protein
CT scan: computed tomography scan
CTD-PAH: connective tissue disease associated with pulmonary hypertension
IL-1: interleukin-1
IPAH: idiopathic pulmonary arterial hypertension
mPAP: mean pulmonary artery pressure
PAH: pulmonary arterial hypertension
