# Supplementary material for: Pulmonary arterial hypertension in adult onset Still’s disease: a case report of a severe complication
Source: BMC Pulm Med. 2016 May 10;16:72. doi: 10.1186/s12890-016-0237-x (PMC4862120; doi:10.1186/s12890-016-0237-x)
Supplement: Additional file 1: Table S1. — Case reports of pulmonary arterial hypertension in adult onset Still’s disease in the literature. (DOC 35 kb) [file 12890_2016_237_MOESM1_ESM.doc]

Additional file 1: Table S1 Case reports of pulmonary arterial hypertension in adult onset Still’s disease in the literature.

|  | Age | Sex | Race | Time until PAH diagnosis (months) | Treatment | Response to Treatment | Outcome |
| --- | --- | --- | --- | --- | --- | --- | --- |
| Zen et al., 1990^3^ | 29 | F | Asian | 60 | NA | Deterioration | NA |
| Chen et al., 2006^4^ | 22 | F | NA | 36 | NA | NA | NA |
|  | 32 | F | NA | 24 | NA | NA | NA |
| Mubashir et al., 2007^5^ | 29 | F | African | 108 | Nifedipine then anakinra | Deterioration | Dead 2 months after Anakinra |
| Eduarda Menezes de Siqueira et al., 2008^6^ | 18 | M | NA | 0 | Indomethacin, prednisone, methotrexate | Normalisation | Alive |
| Campos et al., 2012^7^ | 27 | F | Middle Eastern | 84 | Amlodipin then Anakinra | Poor response to amlodipin and good response to Anakinra | Alive |
| Thakare et al., 2013^8^ | 18 | F | Indian | 7 | NA | NA | NA |
| Robillard et al., 2013^9^ | 36 | F | Asian | 24 | Anakinra | Deterioration | Dead |
| Kadavath et al., 2014^10^ | 38 | F | African | 8 | Anakinra then tocilizumab | Deterioration under anakinar, improvement under tocilizumab | Alive |
